# Maternal transfer of IgA and IgG SARS-CoV-2 specific antibodies transplacentally and via breast milk feeding

**DOI:** 10.1371/journal.pone.0284020

**Published:** 2023-04-06

**Authors:** Mohammad M. Sajadi, Narjes Shokatpour, Madeleine Purcell, Zahra Rikhtegaran Tehrani, Allison Lankford, Allison Bathula, James D. Campbell, Elizabeth Adrianne Hammershaimb, Kristopher B. Deatrick, Casey Bor, Dawn M. Parsell, Colleen Dugan, Andrea R. Levine, Sabrina C. Ramelli, Daniel S. Chertow, Daniel L. Herr, Kapil K. Saharia, George K. Lewis, Alison Grazioli

**Affiliations:** 1 VA Maryland Healthcare Center, Baltimore, MD, United States of America; 2 Institute of Human Virology, Baltimore, MD, United States of America; 3 University of Maryland School of Medicine, Baltimore, MD, United States of America; 4 University of Maryland Medical Center, Baltimore, MD, United States of America; 5 National Institutes of Health, Bethesda, MD, United States of America; Waseda University: Waseda Daigaku, JAPAN

## Abstract

**Background:**

Although there have been many studies on antibody responses to SARS-CoV-2 in breast milk, very few have looked at the fate of these in the infant, and whether they are delivered to immunologically relevant sites in infants.

**Methods:**

Mother/infant pairs (mothers who breast milk fed and who were SARS-CoV-2 vaccinated before or after delivery) were recruited for this cross-sectional study. Mother blood, mother breast milk, infant blood, infant nasal specimen, and infant stool was tested for IgA and IgG antibodies against SARS-CoV-2 spike trimer.

**Results:**

Thirty-one mother/infant pairs were recruited. Breast milk fed infants acquired systemic anti-spike IgG antibodies only if their mothers were vaccinated antepartum (100% Antepartum; 0% Postpartum; P<0.0001). Breast milk fed infants acquired mucosal anti-spike IgG antibodies (in the nose) only if their mothers were vaccinated antepartum (89% Antepartum; 0% Postpartum; P<0.0001). None of the infants in either group had anti-spike IgA in the blood. Surprisingly, 33% of the infants whose mothers were vaccinated antepartum had high titer anti-spike IgA in the nose (33% Antepartum; 0% Postpartum; P = 0.03). Half-life of maternally transferred plasma IgG antibodies in the Antepartum infant cohort was ~70 days.

**Conclusion:**

Vaccination antepartum followed by breast milk feeding appears to be the best way to provide systemic and local anti-SARS-CoV-2 antibodies for infants. The presence of high titer SARS-CoV-2-specific IgA in the nose of infants points to the potential importance of breast milk feeding early in life for maternal transfer of mucosal IgA antibodies. Expectant mothers should consider becoming vaccinated antepartum and consider breast milk feeding for optimal transfer of systemic and mucosal antibodies to their infants.

## Introduction

Breast milk feeding is strongly recommended given its many associated health benefits, particularly those related to the neonatal immune system in the first months of life when infants are at greater risk of infections [1]. SARS-CoV-2 vaccines are currently only available to infants that are at least six months of age in many countries. However, infants can theoretically garner protection through passive immunity via transfer of maternal antibodies [2]. Although COVID-19 vaccination is recommended for pregnant women [3, 4], some may choose vaccination postpartum or not at all. Recent studies suggest pregnant and lactating women develop a successful humoral response to SARS-CoV-2 vaccination [5–7] and that vaccination during pregnancy confers benefit to the infants in terms of the reduction of infants hospitalized with COVID-19 [8]. However, the development of passive immunity to COVID-19 via maternal vaccination remain poorly understood and the acquisition of vaccine induced maternal antibodies in infants has not been well characterized. Given the ability of SARS-CoV-2 IgG maternal antibodies to cross into the fetus antepartum [9, 10], and for breast milk containing SARS-CoV-2 specific antibodies [7, 11, 12] we decided to determine the distribution and fate of SARS-CoV-2 vaccine induced antibodies in mothers and infants within and between antepartum and postpartum pairs.

## Methods

In this cross-sectional study, mother/infant pairs at the University of Maryland Medical Center were recruited. Recritment of mother/infant pairs was done through word of mouth. These included two groups: mothers vaccinated antepartum who breast milk fed (Antepartum group), and mothers who breast milk fed but who were vaccinated postpartum (Postpartum group). The vaccines administered were 2 doses of the Pfizer-BioNTech BNT162b2 or Moderna mRNA-123 (primary series). All sampling was done after maternal completion of the primary vaccine series. Infant breast milk consumption was either from breastfeeding or bottle feeding of expressed breast milk. IgG and IgA SARS-CoV-2 spike trimer antibody levels in maternal blood and milk, as well as infant blood, nose, and stool were determined by ELISA. In addition, ELISA for SARS-CoV-2 nucleocapsid (N) protein IgG was performed on blood samples. Mother/infant pairs testing positive for SARS-CoV-2 N IgG were excluded from our analysis. ELISA was slightly modified from one that was previously published for plasma [13], and cut-offs determined for each site-specific assay. Limit of detection of the IgG plasma, milk, nasal, and stool assays were 1:50, 1:1, 1:500, and 1:1, respectively. Limit of detection of the IgA plasma, milk, nasal, and stool assays were 1:100, 1:1, 1:500, and 1:1, respectively. Samples were run in duplicate, and each positive sample was run at multiple dilutions to determine the end-point titer. The end-point titer was defined as the lowest titer that gave a positive reading. Categorical data was tested using the 2-tailed Fisher’s exact test, with P < .05 considered significant. To calculate half-life (t_1/2_) of IgG in plasma and nasal compartments of the entire cohort and for infants with more than time point, continuous decay (linear regression) was used on log10-transformed data, similar to what has been reported previously [14].

All mothers provided written informed consent; the study was approved by the University of Maryland Baltimore Institutional Review Board. Statistical analysis was performed with GraphPad Prism 5.

## Results

The Antepartum group was comprised of 18 mother/infant pairs and the Postpartum group was comprised of 13 mother/infant pairs. The median age of mothers was 33 years (range 27–37) in the Antepartum group and 34 years (range 27–40) in the Postpartum group. The median gestational age of infants in the Antepartum group was 40 weeks (range 30–41) versus 39 weeks (range 38–41) in the Postpartum group. Vaccination occurred at a median 161 days (range 20 to 285) prior to birth in the Antepartum group and 146 days (range 3 to 326) after birth in the Postpartum group. The median age of infants at sampling was at a median of 167 days (range 15 to 402) after birth in the Antepartum group and 353 days (range 38 to 545) in the Postpartum group. Four infants had sampling performed at two timepoints. One mother in the Antepartum group reported a remote history of COVID-19 prior to vaccination. None of the infants had a history of COVID-19. All mother/infant pairs tested negative for IgG to SARS-CoV-2 N protein and thus had no serologic evidence of recent infection. Additional demographic and clinical characteristics of our cohort are detailed in Table 1.

**Table 1:** Demographic information.

	Antepartum vaccination n = 18	Postpartum vaccination n = 13
Mother age, median (range)	33 (27–37)	34 (27–40)
Mother race		
White, n (%)	17 (94)	12 (92)
Black, n (%)	1 (6)	1 (8)
Asian, n (%)	0 (0)	0 (0)
Mother, history of COVID-19, n (%)	1 (6) *	0 (0)
Vaccine type		
Pfizer-BioNTech BNT162b2, n (%)	9 (50)	6 (46)
Moderna mRNA-1273, n (%)	9 (50)	7 (54)
Vaccine booster (postpartum)		
Pfizer-BioNTech BNT162b2, n (%)	5 (28)	1 (8)
Moderna mRNA-1273, n (%)	3 (17)	0 (0)
Infant gestational age at birth, median (range), weeks	40 (30–41)	39 (38–41)
Infant race		
White, n (%)	16 (87)	11 (85)
Black, n (%)	2 (13)	1 (8)
Asian, n (%)	0 (0)	1 (8)
Infant sex		
Male, n (%)	10 (56)	5 (38)
Female, n (%)	8 (44)	8 (62)
Infant, history of COVID-19, n (%)	0 (0)	0 (0)
Infant sampling age	167 (15 to 402)	353 (38 to 545)
Timing of 1^st^ vaccine to birth in days, median (range)	-169 (-20 to -285)	146 (3 to 326)
Timing of 1^st^ vaccine to infant sampling in days, median (range)	310 (98 to 389)**	199 (35 to 232)
Number of ounces of breast milk intake/day, median (range)	25 (0–35)***	25 (0–30)***

* One mother in the antepartum group had COVID-19 and received vaccine antepartum but was negative for nucleocapsid IgG and thus, included.

** Mother plasma and milk sampling done at same time as infant in Antepartum group. In the postpartum group, mother milk sampling done at same time as infant, but blood sampling done at median of 123 days from vaccine (35 to 137)

*** One of the infants in the antepartum vaccinated stopped breast milk feeding several weeks prior to sampling. Three Infants in the postpartum vaccinated groups discontinued breast milk feeding 2–3 months prior to sampling.

**Fig 1:**
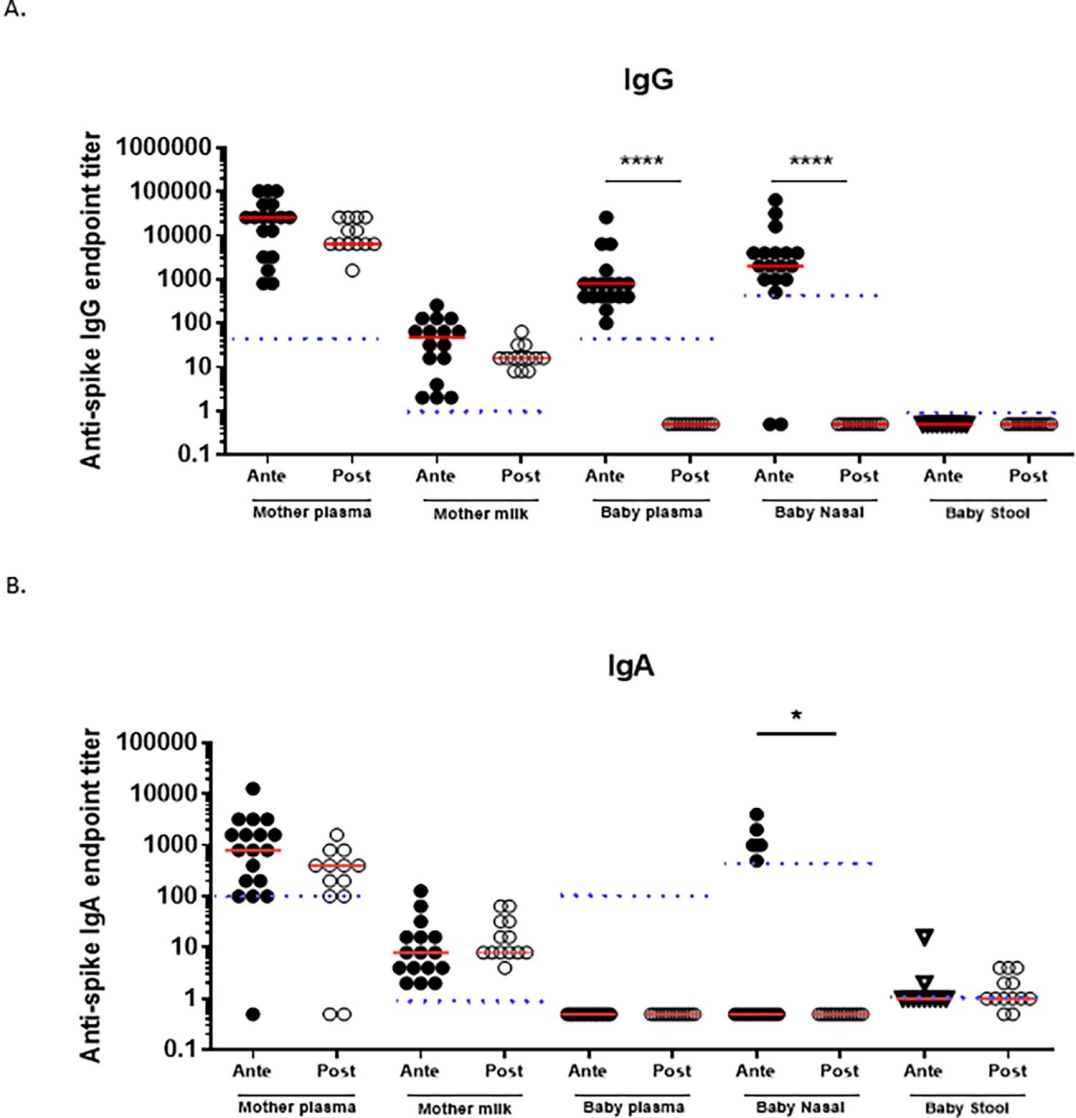
SARS-CoV-2 spike binding titers in vaccinated mothers and infants. A) Log anti-spike IgG binding titer in mother plasma, breast milk, infant plasma, infant nose, and infant stool. Samples below the limited of detection were given an arbitrary value of 0.5. Horizontal red lines represent median values. B) Log anti-spike IgA binding titer in mother plasma, breast milk, infant plasma, infant nose, infant stool. Samples below the limited of detection were given an arbitrary value of 0.5. Horizontal red lines represent medians values, and dotted lines the limit of detection. All ELISAs were designed to detect SARS-CoV-2 spike trimer, samples run in duplicate, and all results reported as endpoint titers. Nasal anti-spike IgG and IgA endpoint titers are reported here as direct results (without providing a ratio with total nasal IgG or IgA). Differences between groups were tested by the 2-tailed Fisher’s exact test, with a P ≤0.05 being considered significant. * = P ≤ 0.05; ** = P ≤ 0.01; *** = P ≤ 0.001; **** = P ≤ 0.0001.

Anti-Spike IgG and IgA were detected in the mothers’ blood and breast milk indicating an appropriate immune response to vaccination (Fig 1A and 1B). There was no difference between antepartum and postpartum vaccinated mothers when comparing SARS-CoV-2 anti-spike protein IgG and IgA in the blood or breast milk (Fig 1A and 1B). All breast milk fed infants (100%) in the Antepartum group had anti-spike IgG in their blood, while none of the breast milk fed infants (0%) in the Postpartum group had anti-spike IgG in their blood (P<0.0001). None of the infants (0%) in either group had anti-spike IgA in the blood.

**Fig 2:**
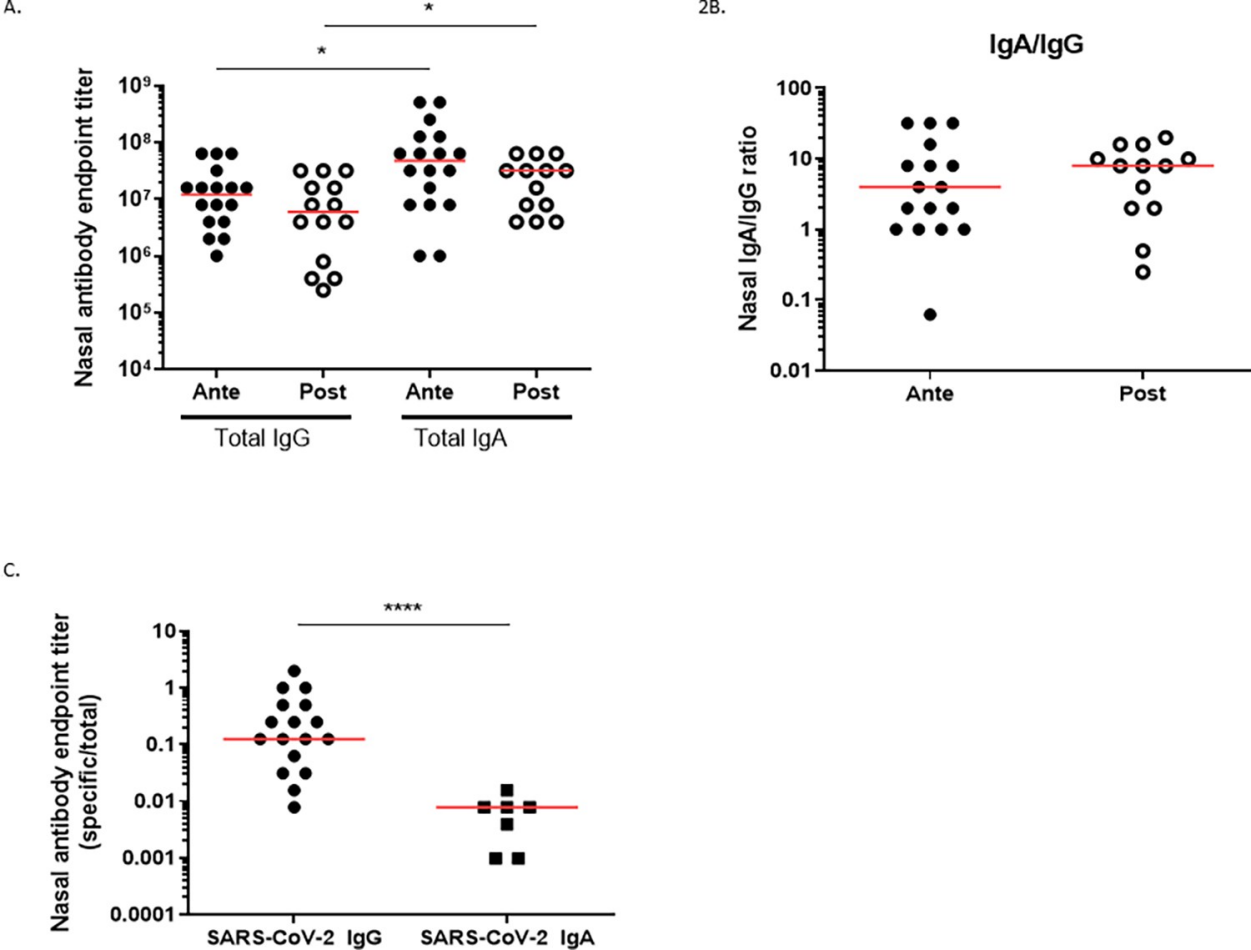
Mucosal humoral immunity in infants. A) IgG and IgA binding titer in infant nose. Horizontal red lines represent median values. B) IgA/IgG endpoint titer in infant nose. Horizontal red lines represent median values. C) Nasal endpoint titers expressed as ratio between anti-spike endpoint titer/Total class endpoint titer. Differences between groups were tested by the 2-tailed Mann Whitney test, with a P ≤0.05 being considered significant. * = P ≤ 0.05; ** = P ≤ 0.01; *** = P ≤ 0.001; **** = P ≤ 0.0001.

All infants had detectable total (non-antigen specific) IgA and IgG in the nasal samples, and no significant differences in total IgG, IgA, or IgA/IgG ratio were noted between the two groups (Fig 2B), though total IgA was higher than total IgG in both groups. Anti-spike IgG was more frequently detected from the nares of breast milk fed infants in the Antepartum group than Postpartum group (89% vs. 0%, P < 0.0001, Fig 1A). P<0.0001) (Fig 1A). Surprisingly, 33% of infants in the Antepartum group had high anti-spike IgA titers in the nares, whereas none of the infants in the Postpartum group had detectable anti-spike IgA in the nares (Fig 1B). The difference in the proportion of infants with detectable anti-spike IgA antibody titers in the nose of the infants in the Antepartum group compared to Postpartum group was statistically significant (P = 0.04). The nasal anti-spike IgA titers (expressed as a ratio between anti-spike IgA titer/total IgA titer) were lower than the nasal anti-spike IgG titers in the infants in the Antepartum group (P = 0.0005) (Fig 2C).

**Fig 3:**
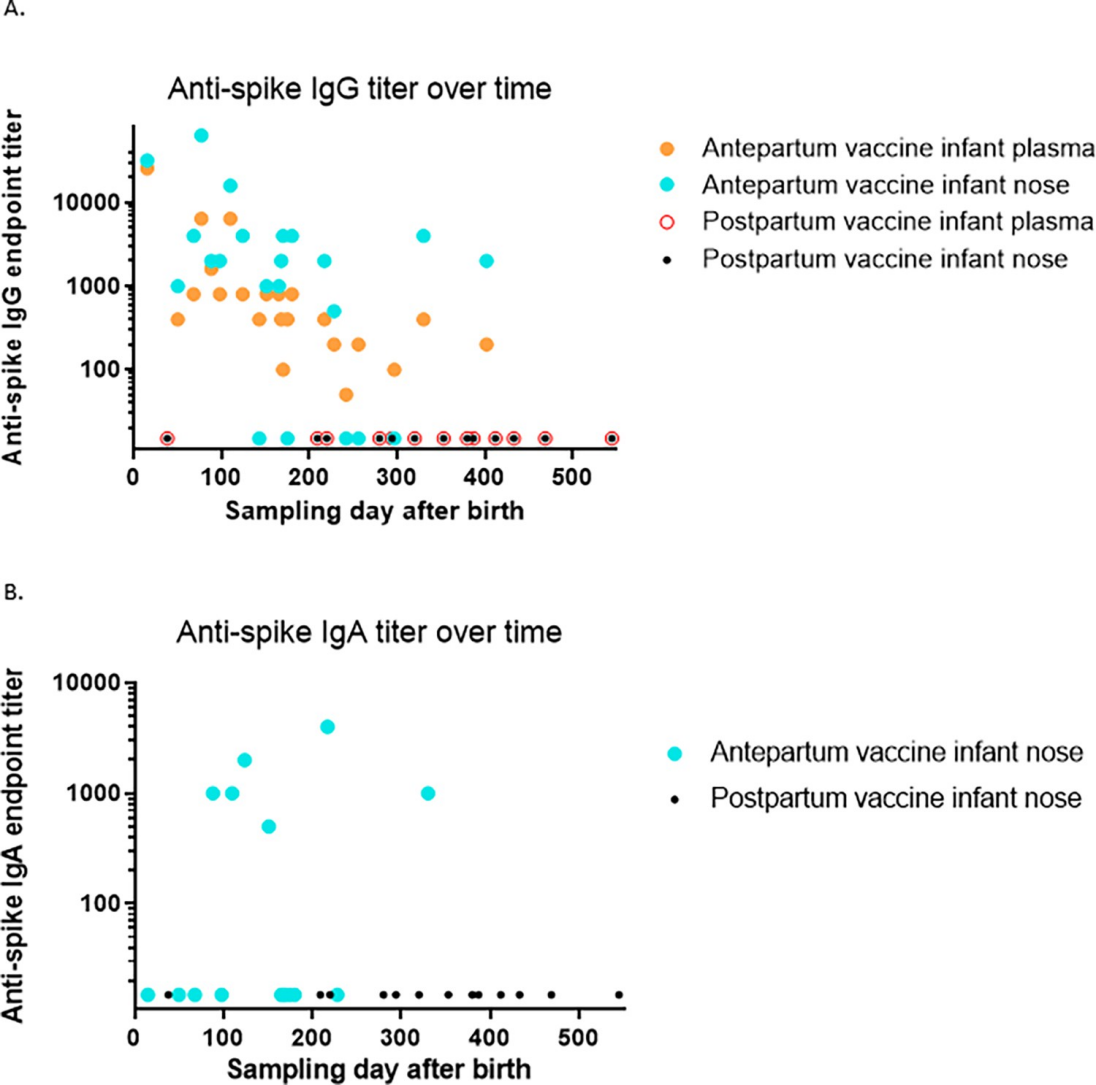
Infant antibodies to SARS-CoV-2 spike antigen over time. A) Infant plasma and nasal anti-spike IgG log transformed and plotted (Y-axis) vs sampling day (X-axis). Samples below the limit of detection were given an arbitrary value of 15. B) Infant nasal anti-spike IgA log transformed and plotted (Y-axis) vs sampling day (X-axis). Samples testing negative were given an arbitrary value of 15. Nasal anti-spike IgG and IgA endpoint titers are reported here as direct results (without providing a ratio with total nasal IgG or IgA).

**Fig 4:**
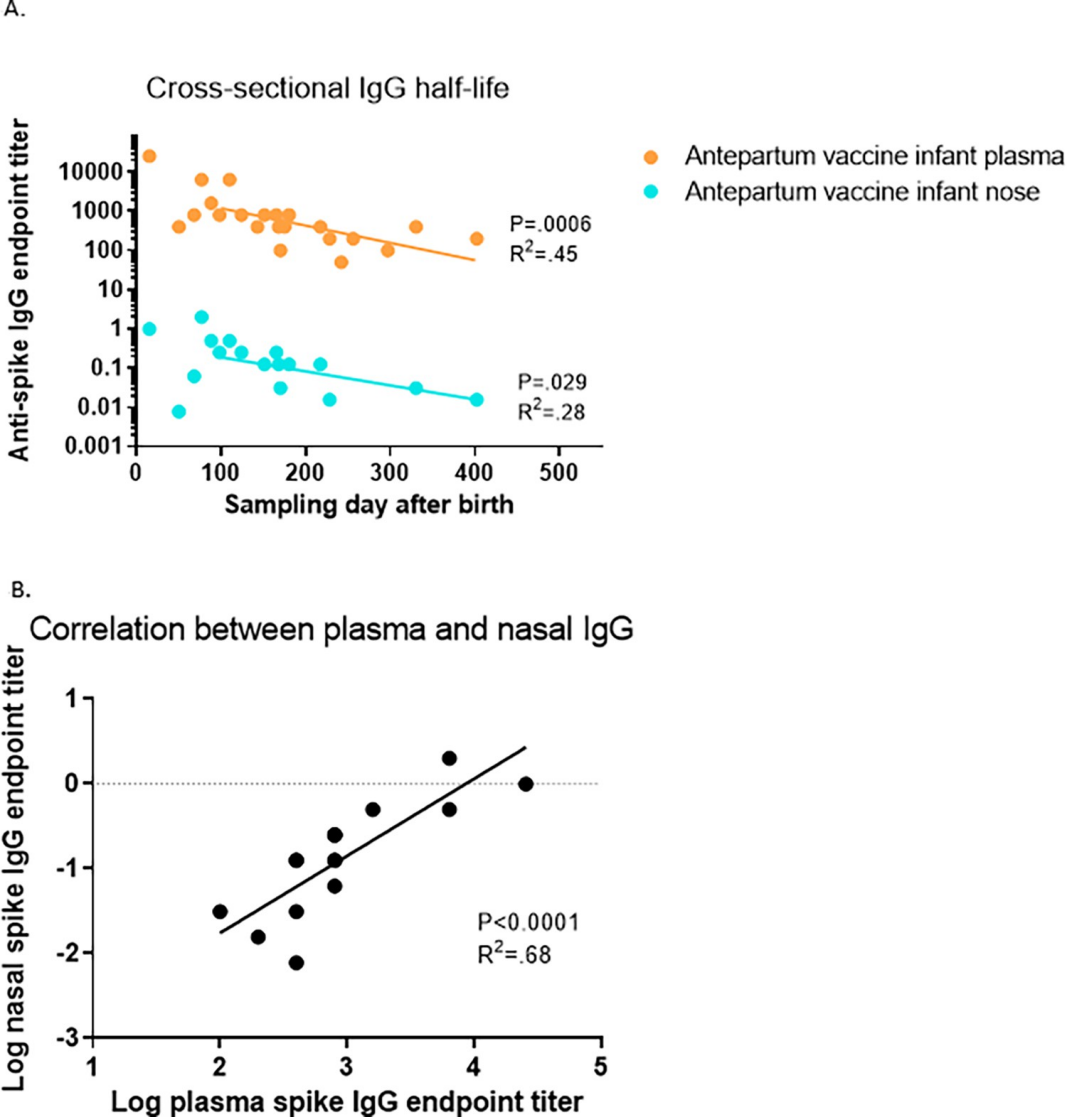
Half-life of maternal IgG in infants and correlation between SARS-CoV-2 spike antibodies nasal and plasma anti-spike IgG. A) Cross-sectional anti-spike IgG from antepartum infant plasma and nasal samples. Using linear regression, cross-sectional plasma IgG t1/2 = 56.9 days; cross-sectional nasal IgG t1/2 = 83.6 days. B) Correlation between plasma and nasal anti-spike IgG endpoint titers. P<0.0001; R^2^ = 0.68. Nasal anti-spike endpoint titers provided as a ratio between nasal IgG endpoint titer/total nasal IgG endpoint titer.

We plotted the nasal and plasma anti-spike IgG and IgA against time (Fig 3A and 3B). Plasma and nasal IgG titers decrease with the increasing age of the infants. Using linear regression, the cross-sectional t_1/2_ of plasma maternal anti-spike IgG in the entire infant cohort was 56.9 days, while the cross-sectional t_1/2_ of nasal maternal anti-spike IgG in the entire infant cohort was 83.6 days (Fig 4A). In 4 of the infants who had detectable anti-spike plasma antibodies, a second sample was obtained 69–72 days after the first sampling, with endpoint anti-spike IgG titers decreasing by a factor of 2 in all the infants. Thus, the t_1/2_ of maternally transferred plasma anti-spike IgG in these 4 infants was ~70 days (10 weeks). There was a direct correlation between the plasma anti-spike IgG and nasal anti-spike IgG spike endpoint titers (P<0.0001; R^2^ = 0.68) (Fig 4B). The nasal IgA data set was not large enough to analyze further.

## Discussion

Transplacental transfer of IgG occurs [15] via the binding of IgG to the neonatal Fc receptor (FcRn) [16]. This was clearly seen in our study, whereby mothers who had SARS CoV-2 anti-spike IgG serum antibodies from vaccination antepartum transferred these antibodies to the blood of their infants. Likewise, mothers who had no anti-spike IgG until postpartum did not transfer SARS-CoV-2-specific IgG to their infants. The half-life of antibody is about twice as long in infants compared to adults [17]. It is noteworthy that IgG was detected in all infants of the Antepartum group (the oldest at 11 months of age), while none was detected in the Postpartum group of breast milk fed infants (as young as 1 month).

In some mammals, infants have the ability to absorb antibodies through the GI tract after delivery. However, studies have shown that this does not occur in human infants, with the exception of pre-mature infants, and the newborn during the first day of life [18]. In one study that delivered oral anti-polio antibody to infants in the first days of life, less than 10% of the antibody was absorbed [18]. After 24 hours of life, none was absorbed [18], and thus this route of antibody transfer (for intact antibody) does not have importance in human infants. The results of the current study are entirely consistent with this finding. Because IgA is not transferred transplacentally, if there was significant absorption of IgA through the GI tract, we would have detected this in blood of the infants in the antepartum vaccination group. However, none of the infants in the antepartum group had detectable anti-spike IgA in the blood. The lack of systemic anti-spike IgG and IgA in the Postpartum infants is also consistent with the literature that GI absorption of antibodies in human infants does not occur. Several mothers in the Postpartum group had stopped breast milk feeding for 2–3 months prior to infant sampling, but given the long half-lives of IgG in the infants, this would only cause a 2–4 fold reduction in the measured titer of circulating antibody in the infant. Thus, if SARS-CoV-2 IgG antibodies had been transferred they would still be detectable.

A strength of our study was the use of endpoint titers, and evaluating all relevant compartments, so they could be directly compared to one another. In the Postpartum group, we found anti-spike IgG and IgA antibodies in the mother’s breast milk and blood. This is consistent with prior studies which have found antibodies, including neutralizing antibody in the breast milk [19, 20]. However, in the Postpartum group, the finding of low anti-spike IgA titers in the stool of infants, absent anti-spike IgG titers in the stool of all infants, and lack of detection of systemic or local antibodies in the infants means that in all likelihood all the anti-spike antibodies consumed in breast milk by the Postpartum infants is being digested in the GI tract without reaching the immunologically relevant sites.

In 1976, there was a seminal study that described protection of infants against RSV that was linked to breast milk feeding, and that this was likely due to IgA [21]. This study has been reproduced and validated multiple times [22–25], but the mechanism by which this occurs has not been completely defined. However, several follow-on studies have shown that in breast milk fed infants, nasal IgA is detected earlier in life than in formula fed infants [26, 27]. This suggests a transfer of IgA from mothers in the breast milk fed infants, while formula fed infants only obtain IgA once the infant starts producing their own. One potential mechanism for this, which has been demonstrated in animals and human infants, is the transfer of immune cells from the mother to infant in the first day of life with ingestion of colostrum [18]. It is known that CD8 cells reactive to PPD have shown to transfer from mothers to human infants (infant will be PPD positive if mother is) [18], and thus anti-SARS-CoV-2 cells, which are present in breast milk, may be transferred as well.

In our study, the most unexpected finding was the presence of high-titer of anti-spike IgA in the nares of infants whose mothers were vaccinated antepartum. This may offer insight into why breast milk feeding can protect against respiratory pathogens. However, the timing of breast milk feeding likely matters, as do other factors, as nasal anti-spike IgA antibody was not found in all infants. None of the infants had SARS-CoV-2 infection by history or serology, thus the only way to transfer the antibodies was via breast milk feeding. This is supported by one prior study in which total IgA was noted to occur in the first weeks of life in half of breast milk fed, but not formula fed newborns [27]. Additionally, it was noted that identical twins had discordant IgA results, suggesting maternal transfer of IgA occurs postpartum and only in some [27].

In light of the studies mentioned above, and the known transfer of IgA by early breast milk feeding, these data are consistent with immune cells being transferred in a percentage of infants early in life. Alternatively, pharyngonasal reflux or direct inhalation could directly deposit the vaccine-specific IgA on nasal surfaces, but another mechanism for concentrating the antibodies (such as binding to mucin or lectin) would need to be involved. Finally, another possibility is the transfer of SARS-CoV-2 antigen from mother to child. Although immune complexes in breastmilk and oral IgA in neonates have been shown in peripartum SARS-CoV-2 infection [28], it is less likely in this case as the median time of vaccination to birth in those with nasal IgA was ~ 6 months (175 days).

As mentioned above the half-life of plasma IgG antibodies in infants is known to be much longer than in adults. This was demonstrated in the 4 infants we had repeated measurements (t_1/2_ ~70 days) and by analyzing the cross-sectional data set as a whole (t_1/2_ = 56.9 days). However, the calculated cross-sectional t_1/2_ of the nasal IgG appeared to be even higher (83.6 days). Although we are not confident in the actual t_1/2_ derived from the plasma and nasal data cross-sectionally, what is interesting is the differing numbers and strength of association. One potential reason for this lack of correlation could be differential regulation of the nasal IgG, with a replenishing source of nasal IgG antibodies after birth, as opposed to plasma IgG which is expected to undergo a steady decay after lack of continuing transfer of antibody after birth. Thus, the findings of high titers of nasal IgG in the infants in the Antepartum group could also be due to the same mechanisms of the transfer of IgA; however, transfer of circulating IgG to mucosal surfaces is also contributing, which could explain the higher percentages of Antepartum infants with detectable nasal IgG compared to IgA, and higher titers of nasal IgG compared to IgA.

Limitations of this study include difference in ages of the infants in the two groups (partly due to vaccine schedule), cross-sectional design, small study population, and small sample volume that precluded functional (neutralization) testing. Although we did look at multiple sites, it is possible that results may be different in other potentially relevant ones not studied (such as lower respiratory tract). Finally, protection of the infant from SARS-CoV-2 was not studied, and postpartum vaccination and protection of the mother could also lead to indirect protection of the infant through cocooning.

## Conclusion

These data suggest that antepartum vaccination followed by breast milk feeding provides the most reliable source of systemic and mucosal antibodies against SARS-CoV-2 for infants. Breast milk fed infants only acquired systemic anti-SARS-CoV-2 antibodies if their mothers were vaccinated antepartum. While breast milk feeding brings many benefits for the infant, there does not appear to be any meaningful transfer of SARS-CoV-2 specific antibody from mothers to their infants if vaccinated postpartum. Expectant mothers should consider becoming vaccinated antepartum and consider breast milk feeding if they wish to transfer SARS-CoV-2 specific antibodies to their infants.

## Supporting information

S1 Data (XLSX)
